# Making sense of missense: challenges and opportunities in variant pathogenicity prediction

**DOI:** 10.1242/dmm.052218

**Published:** 2024-12-16

**Authors:** Ivan Molotkov, Elaine R. Mardis, Mykyta Artomov

**Affiliations:** ^1^ The Steve and Cindy Rasmussen Institute for Genomic Medicine, Abigail Wexner Research Institute at Nationwide Children's Hospital, Columbus, OH 43215, USA; ^2^ Department of Pediatrics, The Ohio State University College of Medicine, Columbus, OH 43215, USA

## Abstract

Computational tools for predicting variant pathogenicity are widely used to support clinical variant interpretation. Recently, several models, which do not rely on known variant classifications during training, have been developed. These approaches can potentially overcome biases of current clinical databases, such as misclassifications, and can potentially better generalize to novel, unclassified variants. AlphaMissense is one such model, built on the highly successful protein structure prediction model, AlphaFold. AlphaMissense has shown great performance in benchmarks of functional and clinical data, outperforming many supervised models that were trained on similar data. However, like other *in silico* predictors, AlphaMissense has notable limitations. As a large deep learning model, it lacks interpretability, does not assess the functional impact of variants, and provides pathogenicity scores that are not disease specific. Improving interpretability and precision in computational tools for variant interpretation remains a promising area for advancing clinical genetics.

## Teaser

**Summary:** This Editorial discusses *in silico* tools for predicting missense variant pathogenicity, with a focus on AlphaMissense, highlighting their strengths, limitations and challenges in unbiased performance benchmarking.

## Computational tools for genetic variant interpretation

Approximately 2-6% of the US population is affected by rare genetic disorders (Ferreira, 2019), making identifying the disease-causing variants critical for guiding treatment and predicting disease progression. Early diagnosis and intervention can also help prevent the irreversible tissue damage often associated with these conditions (Gray, 2016). Although the cost of DNA sequencing has dropped significantly in recent years (Schwarze et al., 2018), effectively pinpointing the pathogenic variation that underlies disease etiology, whether in disease gene discovery studies or diagnostic genetic testing, remains a significant challenge.

There are two key challenges in clinical genetics when diagnosing a specific patient. First, incomplete knowledge of disease genes and causal variants reduces diagnostic yield (Marian, 2020). This underscores the need for more effective methods to identify potentially pathogenic variants and gene–disease associations. Second, even when current knowledge allows for a successful diagnosis, the analysis process remains labor intensive. A recent study by the Medical Genome Initiative found that it takes highly trained experts an average of 7.3 h to analyze a single case (Austin-Tse et al., 2022). As such, streamlining the process for prioritizing pathogenic variants could significantly reduce the resources and time needed for analysis.

*In silico* variant pathogenicity predictors may be used to address these challenges. These computational tools primarily focus on interpreting variants within the coding DNA region. Variants can be different in their ability to affect encoded protein structure and potential to cause pathogenic consequences. For example, synonymous variants do not alter the amino acid sequence of a protein and are generally not expected to impact its function. In contrast, nonsense variants introduce a premature stop codon, leading to a truncated protein that often may lose its function. Unlike these two types, missense variants change a single amino acid and can have diverse effects on a protein, potentially altering its catalytic activity, folding and stability. This diversity makes interpretation of missense variants a challenge and a key target for pathogenicity prediction.

Applications of the pathogenicity predictors are broad across computational and clinical genetics. In association studies comparing genetic variation between cases and controls, these predictors help focus on likely pathogenic variants, reducing noise from neutral variation (Chen et al., 2022). This approach increases statistical power and improves the efficiency of identifying disease-causing genes and variants, further nominating them as diagnostic targets (Cirnigliaro et al. 2023). In the clinical genetics setting, *in silico* tools can narrow down the list of potential causal variants, reducing the workload for analysts. Although they cannot fully replace human experts, these tools can streamline the process by prioritizing variants for review that are most likely pathogenic.

## Supervised and unsupervised pathogenicity predictors

**Fig. 1.**
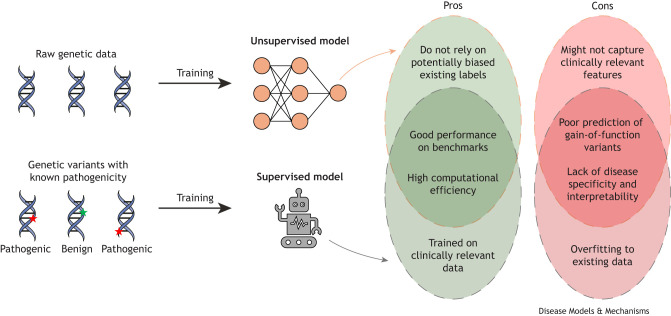
**Comparison of supervised and unsupervised approaches for training models in variant pathogenicity prediction.** Supervised models are trained using variants with expert-assigned pathogenicity labels, whereas unsupervised models rely only on raw DNA or protein sequence data, or weak labels like allele frequencies. Both approaches perform well on clinical and functional datasets, but each has pros and cons: unsupervised approaches are less likely to reproduce biases and errors from labeled data, while supervised approaches may be more directly tailored to predict disease relevance.

Most existing *in silico* predictors are supervised models that utilize variants with known pathogenicity labels to train predictive algorithms (Fig. 1). These models predict pathogenicity using data such as evolutionary conservation, amino acid properties, protein-level characteristics and aggregate predictions from other tools (Katsonis et al., 2022). Large databases like ClinVar (Landrum et al., 2016) and the Human Gene Mutation Database (HGMD) (Stenson et al., 2020) provide hundreds of thousands of clinically classified variants for training. However, relying on labeled data introduces potential biases and limitations, as these databases may contain errors or may not comprehensively represent all genetic variations.

Recently, deep learning-based models that do not use variants with known pathogenicity classification during training emerged as powerful alternatives (Fig. 1). For simplicity, we will refer to these models as ‘unsupervised’, even though some of them can use weak labels like allele frequencies during the training process. Despite not using information on known variant classes, these unsupervised models often outperform many supervised approaches. A notable example is AlphaMissense (Cheng et al., 2023), an unsupervised deep learning model that analyzes raw genomic and protein sequence data. It builds on AlphaFold2 (Jumper et al., 2021) by integrating the detailed three-dimensional structural information of proteins with sequence data and evolutionary conservation insights. By analyzing how a missense mutation might alter the protein's structure and affect its function, AlphaMissense predicts whether the variant is likely to be pathogenic. The advantage of not relying on labeled data is that the biases and limitations associated with current variant pathogenicity databases are obviated. This advantage allows unsupervised models like AlphaMissense to uncover new patterns and relationships that supervised models might overlook owing to their dependence on predefined training labels (Fig. 1).

## Benchmarking AlphaMissense

Both supervised and unsupervised pathogenicity predictors vary in the features they use, their underlying predictive models and training data. To determine which approach is more effective in practice, robust performance evaluations across multiple benchmarks are necessary. Benchmarking *in silico* pathogenicity predictors typically relies on two main data sources: clinical data containing variants that have been accurately classified through genetic analysis, or experimental data from multiplexed assays of variant effect (MAVE) (Starita et al., 2017), which assess the functional impact of variants on a protein using high-throughput *in vitro* methods.

Because AlphaMissense is a relatively new tool, it has not been included in many benchmarking studies. However, a notable exception is the recent evaluation by Livesey et al., which demonstrated AlphaMissense's strong performance across MAVE and clinical data (Livesey and Marsh, 2024 preprint). When benchmarked on MAVE data from 36 proteins, AlphaMissense ranked as the second-best predictor, only outperformed by cross-protein transfer (CPT) (Jagota et al., 2023), another unsupervised model. On clinical datasets, it placed third, following SNPred (Molotkov et al., 2023 preprint) and MetaRNN (Li et al., 2022), but surpassed other unsupervised models.

However, there are some important caveats to consider. The MAVE data used by Livesey et al. for benchmarking the predictors were not validated for concordance with clinical labels. This means that functional scores from the respective MAVE experiments may not accurately reflect variant pathogenicity. Frequent discordance between MAVE experiments and clinically observed variant pathogenicity has been well documented (Gelman et al., 2019). Benchmarking with clinical data has inherent challenges. It includes only resolved variants, which may not represent the full range of patient variants. Expert-assigned labels may contain errors, potentially boosting the performance of tools that replicate these errors during benchmarking. Additionally, tools trained on clinical data may show inflated performance due to information leakage (Livesey and Marsh, 2024 preprint). Despite these limitations, AlphaMissense has consistently shown strong performance across a variety of validation contexts, making it a promising tool in the field of variant pathogenicity prediction.

## Limitations of AlphaMissense

Despite its strong average performance, AlphaMissense has notable limitations that can lead to systematic misclassifications. For example, in one study in which AlphaMissense was applied to 18 experimentally confirmed benign variants in the *IRF6* gene, it misclassified 15 of them as pathogenic, yielding a false positive rate of 83.3% (Murali et al., 2024). The specific reasons for these errors are unclear, partly due to the lack of interpretability in large deep learning models. A similar challenge arose with the *CPA1* gene, for which gain-of-function variants are associated with pancreatitis, but loss-of-function variants are typically benign. Despite the fact that loss of the primary function of CPA1 does not cause the disease, AlphaMissense frequently misclassified variants that reduced the catalytic activity of CPA1 as pathogenic (Wang et al., 2024). Additionally, AlphaMissense classified variants that increased the proteolytic susceptibility of CPA1 as highly pathogenic, despite the irrelevance of this property to disease onset. These errors highlighted the problem of AlphaMissense conflating the impact of a variant on protein function with its actual relevance to disease onset and led researchers to conclude that “AlphaMissense cannot replace wet lab studies as the rate of erroneous predictions is relatively high” (Sándor et al., 2024).

Beyond these specific cases, AlphaMissense shares broader limitations common to many *in silico* predictors. First, AlphaMissense is specifically designed to predict the pathogenicity of missense variants and cannot be applied to other types of genetic variants, such as insertions, deletions, structural variants or variants in non-coding regions. To decide whether a missense variant with a given AlphaMissense pathogenicity score is truly pathogenic, the authors recommend using a single threshold of 0.56. However, this threshold was calibrated only on bulk ClinVar data, so there is no guarantee that it will work equally well across different genes and domains. Additionally, the model does not provide information on the functional effects of variants, offering no insight into the mechanisms driving pathogenicity. Furthermore, the authors of AlphaMissense acknowledge its potentially limited performance in interpreting *de novo* variants, which may lack the evolutionary conservation data necessary for accurate predictions (Cheng et al., 2023).

## Conclusions

AlphaMissense has garnered significant attention, partly due to its association with the highly successful protein folding model, AlphaFold, the inventors of which were recognized with the 2024 Nobel Prize in Chemistry. AlphaMissense demonstrates strong potential in using unsupervised learning techniques to predict the pathogenicity of genetic variants, offering a way to address biases from training on existing data without sacrificing model performance. However, there is still limited evidence that unsupervised models like AlphaMissense offer unique advantages over other approaches in practice.

Although AlphaMissense shows strong performance on current benchmarks, it remains comparable to other state-of-the-art tools and shares many of their limitations. On one hand, it is a reliable tool that provides valuable variant annotations, which can assist genetic analysts and complement other predictors. On the other hand, its impact on variant interpretation has not been as revolutionary as AlphaFold's was for protein folding prediction. We believe that there are several reasons for this difference. Whereas AlphaFold marked an “anomalous leap in protein structure prediction” (AlQuraishi, 2019), AlphaMissense performs similarly to existing predictive models (Livesey and Marsh, 2024 preprint). It also does not present a completely novel approach to pathogenicity prediction because it is not the first model to successfully use a deep protein language model for predictions (Senior et al., 2020). Additionally, variant pathogenicity is influenced by cellular context, environment, epigenetics and more, making it a less well-defined problem than protein folding. Even a complete understanding of a variant's effect on protein structure, function and stability might not be enough to assess pathogenicity without considering these additional factors. Therefore, any approach that tries to infer pathogenicity from protein structure alone may systematically misclassify variants.

Although the field of *in silico* pathogenicity predictors is steadily growing, several key aspects of variant interpretation remain underexplored. Most of the current methods focus primarily on missense variants, and the pathogenicity predictions they generate are often difficult to interpret. Moreover, only a few models incorporate disease-specificity information during training [such as CAVaLRi (Schuetz et al., 2024)], whereas the majority predict pathogenicity in a more general sense that might not correspond to the patient's actual phenotype. Consequently, even if a variant is correctly classified as pathogenic, it can be clinically misleading if it does not correspond to the patient's actual phenotype. To enhance clinical relevance, the field of computational variant effect prediction should shift toward creating more interpretable models that assess variant impact in the context of specific patient phenotypes.
